# Outcomes and financial implications of intra-articular distal radius fractures: a comparative study of open reduction internal fixation (ORIF) with volar locking plates versus nonoperative management

**DOI:** 10.1007/s10195-016-0441-8

**Published:** 2017-02-02

**Authors:** Dong Hao Toon, Rex Antony Xavier Premchand, Jane Sim, Rajaratnam Vaikunthan

**Affiliations:** 1 Orthopaedic Surgery, Khoo Teck Puat Hospital, 90 Yishun Central, Singapore, 768828 Singapore; 2 Rehabilitation Services, Khoo Teck Puat Hospital, 90 Yishun Central, Singapore, 768828 Singapore

**Keywords:** Adult, Cast, Surgical, Fracture fixation, Health care costs, Intra-articular fractures, Radius fractures, Range of motion, Wrist joint

## Abstract

**Background:**

To evaluate the functional and radiographic outcomes, as well as the treatment costs, of closed displaced intra-articular distal radius fractures treated with either open reduction internal fixation (ORIF) with volar locking plates or nonoperative treatment with plaster cast immobilisation.

**Materials and methods:**

A total of 60 patients (32 receiving ORIF, 28 receiving nonoperative treatment) with closed intra-articular distal radius fractures were included. The mean age was 52.1 and 57.4, respectively. Functional and radiographic assessments were carried out at 12 months post-injury. Patients’ treatment costs, median salaries and lengths of medical leave were obtained.

**Results:**

DASH and MAYO wrist score in the ORIF group did not differ significantly from those in the nonoperative group. Apart from superior ulnar deviation in the ORIF group (*p* = 0.0096), differences in the range of motion of the injured wrists were not significant. Similarly, there were no significant differences in grip strength and visual analog scale for pain. Volar tilt (*p* = 0.0399), radial height (*p* = 0.0087), radial inclination (*p* = 0.0051) and articular step-off (*p* = 0.0002) were all significantly superior in the ORIF group. There was a 37-fold difference in mean treatment costs between ORIF (SGD 7951.23) and nonoperative treatment (SGD 230.52).

**Conclusion:**

Our study shows no difference in overall functional outcomes at 12 months for closed displaced intra-articular distal radius fractures treated with either ORIF with volar locking plates or plaster cast immobilisation, and this is independent of radiographic outcome. A longer follow-up, nevertheless, is needed to determine whether the development of post-traumatic arthritis will have an effect on function. The vast difference in treatment costs should be taken into consideration when deciding on the treatment option.

**Level of evidence:**

Level 3.

## Introduction

Fractures of the distal radius are among the most common orthopaedic injuries, and impose a significant financial burden on healthcare [1]. However, given the prevalence of distal radius fractures, controversy remains concerning the best management. Although several surgical options are available, including percutaneous pinning, external fixation, open reduction internal fixation (ORIF) techniques, intramedullary fixation, as well as arthroscopic assisted reduction and fixation, the 2009 American Academy of Orthopaedic Surgeons (AAOS) clinical practice guideline (CPG) was unable to recommend for or against any one specific surgical method [2]. Despite this lack of consensus, ORIF of distal radius fractures has become increasingly popular in recent years, particularly in relation to the use of volar locking plates [3–5]. With the rise in cost-consciousness in our healthcare system, it is important to determine the outcomes and financial implications of treating these fractures with this surgical method. Our study aims to evaluate the functional and radiographic outcomes at 12 months, as well as the treatment costs, of closed displaced intra-articular distal radius fractures treated with ORIF with volar locking plates versus nonoperative treatment with immobilisation in plaster cast.

## Materials and methods

This study was conducted in Khoo Teck Puat Hospital (KTPH), Singapore. Approval from the local research ethics board was granted prior to initiation of the study. All patients provided written informed consent for their participation.

Between 1st January 2011 and 1st January 2012, a total of 196 patients who presented to KTPH with distal radius fractures were extracted from the hospital database and evaluated for eligibility. Inclusion criteria were adult patients (aged 21 years and above) with a closed displaced intra-articular distal radius fracture (classified as AO group B and group C). On presentation, patients underwent closed manipulation and reduction under sedation. A below-elbow partial cast was applied and post-reduction radiographs were obtained. Patients were counselled on treatment options of ORIF and nonoperative treatment and the decision was made based on patient autonomy. Patients in the ORIF group underwent ORIF using the Depuy Synthes 2.4-mm variable-angle LCP volar distal radius plate. For patients in the nonoperative group, the partial cast was converted into a below-elbow plaster of Paris complete cast within a week. The complete cast was removed at 4 weeks and fractures were assessed for clinical and radiographic union. Fractures that were deemed not united were continued in the complete cast for an additional 2 weeks (total of 6 weeks). Rehabilitation was initiated by the hand occupational therapist the day after surgery for the ORIF group, and on the same day as the cast was removed in the nonoperative group. Patients with ipsilateral upper limb fractures, pathological fractures, open fractures, a delay in presentation of more than 14 days, or those who did not follow the hand occupational therapy protocol following surgery or plaster cast immobilisation were excluded from our study.

Of the 87 patients who met the inclusion and exclusion criteria, 27 were lost to follow-up or did not give consent for participation, resulting in a final number of 60 patients (32 operative, 28 nonoperative). The mean age was 52.1 in the ORIF group and 57.4 in the nonoperative group. The male to female ratio was 14:18 and 11:17 in the respective groups. 46.88% of those in the ORIF group sustained fractures on their dominant wrist, whereas 42.86% in the nonoperative group injured their dominant wrists. In the ORIF group, 10 patients (31.25%) had AO group B fractures and 22 patients (68.75%) had AO group C fractures. In the nonoperative group, 6 patients (21.43%) had AO group B fractures and 22 patients (78.57%) had AO group C fractures. A summary of study population demographic and injury characteristics is shown in Table 1.

**Table 1:** Patient demographics and injury characteristics

	Operative (ORIF)	Nonoperative	*p* value
Number of patients	32	28	
Gender			
Male	14	11	0.726
Female	18	17	
Mean age (years)	52.1 (23–77)	57.4 (26–79)	0.146
Dominant side			
Right	29	24	
Left	3	4	
Injured side			
Right	14	12	
Left	18	16	
Dominant side injured			
Yes	15	12	0.755
No	17	16	
AO fracture classification			
B (Total)	10	6	0.391
B1	3	4	
B2	1	1	
B3	6	1	
C (Total)	22	22	
C1	11	12	
C2	6	8	
C3	5	2	

Patients were assessed at 12 months post-injury for functional and radiographic assessments carried out in the outpatient setting. Functional outcomes were determined by using the disabilities of the arm, shoulder, and hand (DASH) score and the MAYO wrist score. Active range of movement of the injured wrist was measured using a goniometer. Grip power was measured using a dynamometer and compared to the noninjured (normal) side. A visual analog scale (VAS) was used to assess pain.

Radiographic measurements were determined by two assessors based on radial inclination, radial height, volar tilt and articular step-off on standard PA and lateral wrist radiographs.

Total treatment costs for both groups were obtained from the hospital finance department. Total outpatient costs comprised the fee incurred from accident to emergency consultation, plaster casts, radiological investigations, outpatient clinic appointments and physiotherapy sessions. For patients in the ORIF group, total inpatient costs including costs of surgery (including surgical implants) and inpatient hospital admission were attained. The patients’ occupations were recorded and the total number of days of medical leave given was obtained from hospital medical records. The estimated financial impact on the economy was determined by calculating the patients’ median salaries (in accordance with the Singapore Ministry of Manpower Median Gross Wages of Common Occupations By Industry, June 2013) [6] multiplied by the number of days of medical leave given.

A power analysis was performed based on the wrist function outcome scores utilised. Categorical data were compared using Pearson’s chi-square test and continuous data were compared using the Mann–Whitney test for nonparametric data and the independent *T* test for parametric data. Statistical significance was assumed when *p* < 0.05.

## Results

**Fig. 1:**
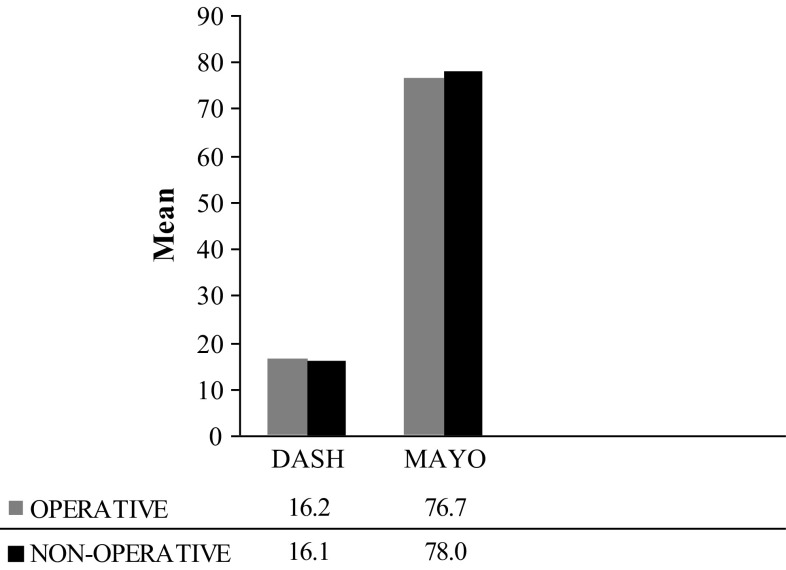
Mean DASH score and Mayo wrist score at 12 months post-injury

The functional and radiographic results are summarised in Table 2. At 12 months post-injury, the DASH score in the ORIF group (16.2 ± 17.4) did not differ significantly from that in the nonoperative group (16.1 ± 17.7) (*p* = 0.9878). Similarly, the MAYO wrist score in the ORIF group (76.7 ± 12.7) did not differ significantly from that in the nonoperative group (78.0 ± 8.6) (*p* = 0.6449) (Fig. 1). There was also no significant difference in the grip strength between the two groups (*p* = 0.6778), or in the visual analog score for pain (*p* = 0.5321).

**Table 2:** Outcomes at 12 months post-injury

	Operative (ORIF)	Nonoperative	*p* value
DASH score	16.2 ± 17.4	16.1 ± 17.7	0.9878
MAYO wrist score	76.7 ± 12.7	78.0 ± 8.6	0.6449
Strength (% compared to contralateral side)	83.29 ± 14.1	81.26 ± 22.9	0.6778
Pain on visual analog scale (VAS)	1.8 ± 1.6	1.1 ± 1.1	0.5321
Range of motion			
Extension (°)	67.5 ± 13.7	72.9 ± 13.2	0.1288
Flexion (°)	63.1 ± 10.2	64.1 ± 13.0	0.7445
Ulnar deviation (°)	22.8 ± 8.0	17.9 ± 6.0	0.0096
Radial deviation (°)	15.6 ± 7.3	15.7 ± 5.2	0.6449
Radiographic parameters			
Volar tilt (°)	5.6 ± 8.9	0.1 ± 11.6	0.0399
Radial height (mm)	9.6 ± 3.3	7.2 ± 3.4	0.0087
Radial inclination (°)	21.6 ± 6.1	16.9 ± 6.3	0.0051
Articular step-off (mm)	0.71 ± 0.58	1.50 ± 0.93	0.0002

**Fig. 2:**
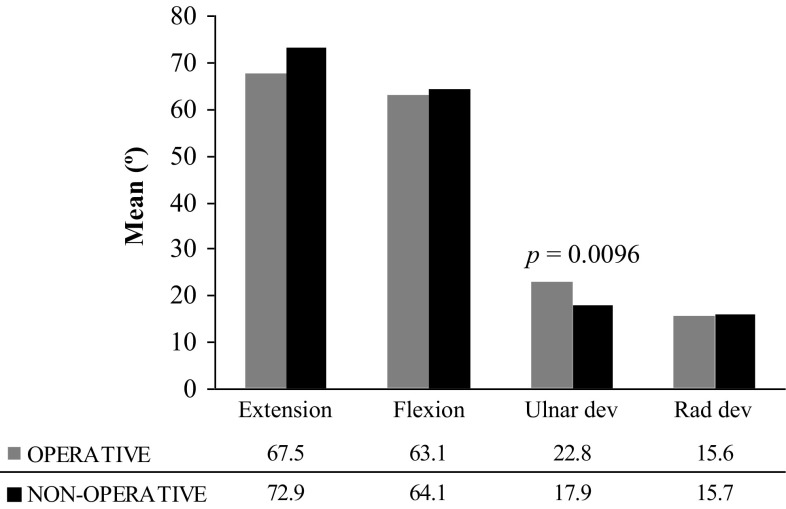
Mean range of motion of injured wrist at 12 months post-injury

For range of motion of the injured wrist, ulnar deviation was significantly superior in the ORIF group (*p* = 0.0096); whereas the differences in extension, flexion and radial deviation did not reach statistical significance (Fig. 2).

**Fig. 3:**
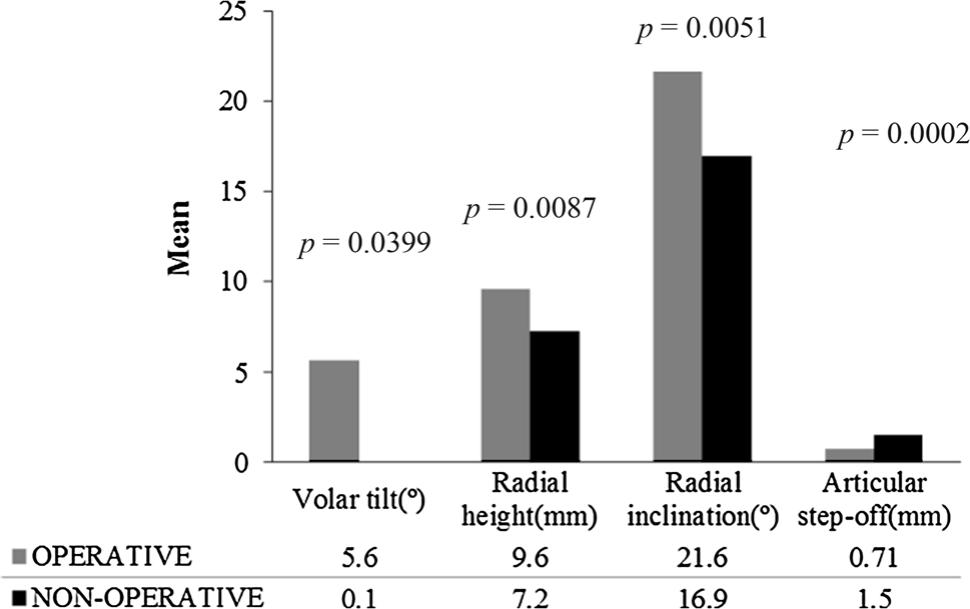
Mean radiographic parameters of the distal radius at 12 months post-injury

In contrast, the ORIF group was significantly superior in all radiographic parameters at 12 months post-injury, including radial height (*p* = 0.0087), radial inclination (*p* = 0.0051), volar tilt (*p* = 0.0399) and articular step-off (*p* = 0.0002) (Fig. 3).

One patient in the ORIF group underwent removal of implants due to tendon irritation. There were no other implant-related complications such as infection, tendon rupture, intra-articular screw penetration, and wound healing problems.

The mean number of days of medical leave given was 85 ± 87 for the ORIF group and 46 ± 38 for the nonoperative group (*p* = 0.066). (Five patients in the ORIF group and seven patients in the nonoperative group were unemployed, self-employed or retired and therefore did not require medical leave and were excluded.) A 37-fold difference in the mean treatment costs was found between ORIF (SGD 7951.23 ± 6774.94) and nonoperative treatment (SGD 230.52 ± 113.72). The estimated financial impact on the economy was SGD 140,192.57 in total for the ORIF group and SGD 55,029.14 in total for the nonoperative group.

## Discussion

In our study, ORIF of intra-articular distal radius fractures with the volar locking plate produced statistically significant superior radiographic parameters in volar tilt, radial inclination, radial height and articular congruity at 12 months post-injury compared to nonoperative treatment. However, apart from improved ulnar deviation in the ORIF group, the superiority in radiographic parameters does not translate to better functional outcomes in the DASH and MAYO wrist scores, strength, pain score and range of motion (flexion, extension and radial deviation). In this context, the importance of ulnar deviation has recently been observed by Tsitsilonis et al. [7], who found that limitation of ulnar abduction correlated with inferior quality of life in many subcategories of the SF-36 health survey, which was not included in our study. This correlation could be due to a number of affected activities of daily living that require ulnar deviation, including (1) typing, specifically when pressing the “Enter” key on the keyboard, (2) reaching for an item in one’s pocket, and (3) opening a bottle. Further studies to examine the adequacy of compensatory movement of the elbow and shoulder to accommodate for the reduction in ulnar deviation when performing these actions would be helpful.

The AAOS CPG concluded that for patients aged >55 years old, the available evidence does not demonstrate any difference between casting and surgical fixation. This is supported by several studies which have shown that fracture reduction and anatomic alignment for unstable distal radius fractures in the elderly does not correlate with better functional outcomes as compared with younger patients [8–12]. The ORCHID study [13], the largest multicentre randomised study to date comparing ORIF and nonsurgical treatment of AO type C distal radius fractures in the elderly, showed that differences in wrist joint function and health-related quality of life were not statistically significant. Given the continued rise of the elderly as a proportion of the overall population in most developed countries, more attention must be paid to this group of patients. The potential gap between their chronological and physiological ages should be considered when managing distal radius fractures, as they increasingly remain more active and healthy.

Notably, a much earlier study by Trumble et al. [14], looking at 43 patients with intra-articular distal radius fracture across all age groups (mean age 37, range 17–79), found that although the restoration of articular congruity and correction of radial shortening leads to improve functional outcomes, the correction of radial tilt or dorsal tilt does not. Similar to our study, this was a retrospective study which included patients across a wide age range, but it did have a longer mean follow-up time of 38 months.

Another study which included all adult patients similar to ours, but with a lower mean age of 39, was a randomised controlled trial (RCT) comparing ORIF to casting (19 and 23 patients, respectively) for intra-articular fractures [15]. They found a higher likelihood of excellent function with ORIF than with closed reduction [risk ratio (RR) 0.69, 95% confidence interval (CI) 0.48–1.00]. However, the measure of functional outcomes was not clearly stated, with patients grouped into either “excellent”, “good”, “fair” or “poor” based on “residual deformity, subjective evaluation, objective evaluation and complications”.

The expenditure on treating distal radius fractures is expected to rise with the increasing popularity of ORIF of distal radius fractures. Few studies have directly compared the costs of different treatment options of distal radius fractures. Farner et al. [16] showed that open treatment is associated with higher costs and subsequent complications compared with percutaneous fixation in the elderly. Likewise, in their economic evaluation within a RCT, Karantana et al. [17] found no evidence to support the cost-effectiveness, from the perspective of the National Health Service (NHS), of fixation using a volar locking plate over percutaneous fixation for dorsally displaced distal radial fractures. An economic analysis by Shauver et al. [18] found that although ORIF was the most preferred treatment, it was also the most expensive, compared to casting, wire fixation and external fixation.

A few limitations of our study should be noted. Firstly, this was a retrospective study. Secondly, there was no randomisation of treatment groups. Thirdly, only short-term outcomes (12 months) were measured in this study. Although the sample sizes in the ORIF and nonoperative groups (32 and 28, respectively) after the criteria were applied were by no means small, a larger—ideally prospective and randomised—trial looking into both the short- and long-term outcomes will provide more information and a higher level of evidence. One notable omission from our radiographic assessment was radiographic changes of osteoarthritis. This was due to the perceived short duration (12 months) from injury when radiographic assessment was performed in our study. In addition, early radiographic changes do not always progress and result in clinically relevant post-traumatic osteoarthritis [19, 20].

In conclusion, our study shows that there is no difference in overall functional outcomes between patients with a closed displaced intra-articular distal radius fracture treated with ORIF with volar locking plates and patients treated nonoperatively at 12 months, and this is independent of the superior radiographic outcomes in the ORIF group. The vast difference in the financial costs of treatment should be taken into consideration when deciding on the treatment option.
